# Effects of Hypomagnetic Field Environment on Tumor Progression and Gut Microbiota in LLC Tumor-Bearing Mice

**DOI:** 10.3390/biology15161349

**Published:** 2026-08-10

**Authors:** Wenqi Ti, Detian Zhang, Xiaolin Ning

**Affiliations:** 1 Key Laboratory of Ultra-Weak Magnetic Field Measurement Technology, Ministry of Education, School of Instrumentation and Optoelectronic Engineering, Beihang University, Beijing 100191, China; by2243140@buaa.edu.cn (W.T.); bhzhangdt@buaa.edu.cn (D.Z.); 2 Hefei National Laboratory, Hefei 230088, China; 3 Department of Dermatology, Hunan Engineering Research Center of Skin Health and Disease, Xiangya Hospital, Central South University, Changsha 410008, China

**Keywords:** hypomagnetic field, Lewis lung carcinoma, tumor growth, gut microbiota, short-chain fatty acids

## Abstract

The tumor microenvironment interacts bidirectionally with systemic host factors, including the gut microbiota. However, it remains unclear whether physical environmental stimuli, such as a hypomagnetic field (HMF), can modulate tumor progression and associated microbial features. In this study, C57BL/6 mice bearing subcutaneous Lewis lung carcinoma (LLC) were maintained under geomagnetic field (GMF) or HMF conditions. We evaluated tumor growth and systemic toxicity, and analyzed fecal samples using 16S rRNA sequencing and short-chain fatty acid (SCFA) profiling. Compared with the GMF control, HMF exposure significantly reduced tumor growth, with an approximately 40% decrease in endpoint tumor volume. No obvious systemic toxicity was detected by monitoring body weight, serum biochemical parameters, and organ histology. HMF exposure altered gut microbial diversity and community composition, with enrichment of several Clostridia-related lineages. Fecal SCFA analysis revealed increased butyrate levels in the HMF group (*p* = 0.013). Overall, these findings indicate that HMF exposure is linked to reduced LLC tumor growth and changes in gut microbial composition and metabolites. Further studies are needed to clarify the mechanisms underlying these interactions.

## Simple Summary

Hypomagnetic field (HMF) refers to an environment with magnetic field intensity substantially lower than that of the geomagnetic field. Since living organisms are constantly exposed to the Earth’s magnetic field, deviations in magnetic environments may influence physiological functions and disease-related processes. Here, we established a near-zero magnetic field environment (~20 nT) and investigated its effects on Lewis lung carcinoma (LLC) growth, gut microbial composition, and fecal short-chain fatty acid (SCFA) profiles in tumor-bearing mice. The results showed that HMF exposure suppressed Lewis lung carcinoma progression without causing obvious systemic toxicity. It also altered gut microbial diversity and enriched bacterial groups related to short-chain fatty acid metabolism. Among the detected short-chain fatty acids, butyric acid significantly increased under HMF exposure. These observations indicate that slowed tumor growth under HMF exposure was accompanied by changes in gut microbiota and microbial metabolite profiles. This study provides initial evidence linking altered magnetic environments with tumor biology and host-associated gut microbial responses.

## 1. Introduction

Lung cancer remains a major contributor to global cancer mortality, even with continued progress in clinical diagnosis and therapy [1]. In addition to intrinsic oncogenic alterations, tumor progression is increasingly recognized to involve systemic host factors, such as metabolism, immune regulation, and the gut microbiome [2]. Among these systemic regulators, the gut microbiota has emerged as a key regulator of immune homeostasis and tumor-associated inflammation [3,4]. Clinical studies have shown that antibiotic exposure can attenuate the efficacy of PD-1 immunotherapy, while specific gut microbiota features are associated with superior treatment response [5,6]. Furthermore, animal models have demonstrated that microbial dysbiosis can alter the tumor microenvironment and its growth dynamics [7].

The gut microbiota is sensitive to the external environment. Dietary patterns can induce rapid and reproducible changes in both the taxonomic structure and functional profiles of the human microbiome within short periods [8]. Similarly, antibiotic exposure triggers a precipitous decline in microbial diversity and profound compositional shifts within days, often resulting in incomplete long-term recovery [9]. These observations suggest that interactions among the environment, the microbiome, and the tumor may contribute to heterogeneity in tumor phenotypes and therapeutic responses. However, compared to chemical and biological factors including diet and drugs, the impact of physical environmental variables such as the magnetic environment remains poorly studied.

The geomagnetic field has always constituted a stable physical foundation throughout the entire process of biological evolution. Hypomagnetic field (HMF) environments, defined by magnetic field intensities significantly below the natural geomagnetic background, are typically encountered in deep-space missions, magnetically shielded facilities, and specific controlled experimental settings. Recent studies have shown that exposure to HMF affects oxidative stress, cell proliferation, and intestinal microbial homeostasis in rodents [10]. Meanwhile, prior studies have observed that weak magnetic field conditions at approximately the hundred-nanotesla scale can alter tumor cell adhesion, migration, and actin cytoskeleton [11]. However, at more extreme ultra-weak field levels, the impact on tumor progression remains poorly defined. It is also unclear whether such exposure is accompanied by systematic shifts in the host’s gut microbiota.

In this study, we used a subcutaneous syngeneic Lewis lung carcinoma (LLC) tumor model to examine how HMF exposure affects tumor growth and selected systemic toxicity-related indicators in C57BL/6 mice. 16S rRNA sequencing and fecal short-chain fatty acid (SCFA) profiling were used to assess changes in gut microbial composition and fecal metabolites. Together, these analyses characterized HMF-associated changes in tumor growth, gut microbiota, and fecal SCFA profiles, providing a foundation for further investigation of microbiota-related mechanisms in HMF-associated tumor responses.

## 2. Materials and Methods

### 2.1. Experimental Animals

Female C57BL/6 mice (6 weeks old) were obtained from Liusha Biological Technology Co., Ltd. (Hangzhou, China). The mice were housed under standard conditions with a 12 h light/dark cycle and free access to chow and water. All procedures were approved by the Institutional Animal Care and Use Committee of Xiangya Hospital, Central South University (approval no. XY20250609007).

### 2.2. Cell Culture and Tumor Implantation

Lewis Lung Carcinoma (LLC) cells (Baidi, Hangzhou, China) were cultured in Dulbecco’s modified Eagle’s medium (DMEM; Biochannel, Nanjing, China) containing 10% fetal bovine serum (FBS; Biochannel, Nanjing, China) and 1% penicillin/streptomycin (Biosharp, Hefei, China). Cells were incubated at 37 °C with 5% CO_2_ and passaged when confluence reached 80–90%. After washing with phosphate-buffered saline (PBS; Biosharp, Hefei, China), the cells were detached with 0.25% trypsin–EDTA (Biochannel, Nanjing, China). The enzymatic activity was neutralized with complete culture medium, and cells were passaged at a split ratio of 1:3 or 1:4. For tumor implantation, logarithmically growing LLC cells were collected and washed with serum-free medium. After induction of anesthesia with 3% isoflurane, each mouse received a subcutaneous injection of 1 × 10^6^ LLC cells (100 μL) into the right flank.

### 2.3. Animal Enrollment and HMF Exposure

Before randomization and exposure, tumor size and general health were checked in all mice. Mice were enrolled when tumor volumes ranged from 50 to 100 mm^3,^ and no obvious body weight loss was observed. Sample size was estimated using G*Power software (version 3.1) based on preliminary endpoint tumor volume measurements. The preliminary tumor volumes were 1308.0 ± 112.5 mm^3^ in the GMF group and 1030.7 ± 156.4 mm^3^ in the HMF group. Assuming a two-sided significance level of 0.05 and 80% statistical power, the estimated minimum sample size was five tumor-bearing mice per group. Considering possible exclusions before randomization, 20 mice were initially inoculated with LLC cells. Fourteen mice met the inclusion criteria and were randomly assigned to the geomagnetic field (GMF) control group or the hypomagnetic field (HMF) exposure group using a random number table. Seven mice were included in each group, while six mice were excluded before randomization because they did not meet the enrollment criteria.

The mice were exposed to the corresponding magnetic field conditions for 10 consecutive days. The GMF group remained under normal geomagnetic conditions (~50 μT). The HMF group was maintained in a magnetic shielding chamber with a residual field of approximately 20 nT. Each group was housed in two cages with three to four mice per cage, and cage density was matched between groups. Both groups of mice were maintained in the same room. Cage positions were randomized and rotated regularly to reduce potential cage-related and location-related effects on gut microbiota.

### 2.4. Magnetic Field Characterization and Environmental Control

The HMF setup comprised a multi-layer magnetic shielding box (Figure S1). Before residual-field measurements, the shielding chamber was degaussed using an electromagnetic demagnetization procedure. The residual magnetic field was then measured using a Mag-03 three-axis fluxgate magnetometer (Bartington Instruments Ltd., Witney, UK). The X-, Y-, and Z-axis magnetic-field components were recorded at each measurement position, and the total residual field was calculated as B = $\surd (Bx^{2}+By^{2}+Bz^{2})$. Field stability was checked before exposure and after completion of exposure. Measurements were obtained at 30 locations arranged in a 6 × 5 grid inside the shielding chamber. The mapped area included the full cage area used during exposure. Measurement positions ranged from −50 to 0 cm along the X-axis and from −40 to 0 cm along the Y-axis. The residual field value (~20 nT) was reported based on the maximum measured field after rounding. Illumination was provided by a non-magnetic light source inside the chamber. Light intensity was measured using a Fluke 941 light meter (Fluke Corporation, WA, USA). Both GMF and HMF groups followed the same light/dark cycle. Temperature and relative humidity were maintained at 23 ± 1 °C and 50 ± 2%, respectively. The chamber allowed continuous air exchange to reduce the risk of hypoxia and heat buildup.

### 2.5. Tumor Growth Assessment and Systemic Toxicity Evaluation

During the exposure period, mice were checked regularly for health status, activity, body weight, tumor growth, and signs of distress. Humane endpoints were established before the experiment and included excessive tumor burden, tumor ulceration or necrosis, severe body weight loss, impaired mobility, or other signs of severe distress. No unexpected adverse events were observed during the experiment.

Tumor length (L) and width (W) were recorded periodically using digital calipers. Tumor volume was estimated according to the formula V = L × W^2^ × 0.5. Body weight was recorded at the same time points. At the endpoint, blood samples were collected from the orbital venous plexus under deep anesthesia. Approximately 200 μL of blood was collected from each mouse into EP tubes without anticoagulant. Blood samples were kept at 4 °C for 30 min and then centrifuged to obtain serum. Serum samples were transported on dry ice to Dr.Can Bioscience (Hangzhou, China) for biochemical analysis. Serum biochemical parameters, including alanine aminotransferase (ALT), aspartate aminotransferase (AST), blood urea nitrogen (BUN), and creatinine (CRE), were measured.

Following blood collection, mice were euthanized by cervical dislocation under deep anesthesia. Tumors were excised, photographed, and weighed. The heart, liver, spleen, lung, and kidney were collected and fixed in 4% paraformaldehyde. Fixed tissues were processed through graded ethanol dehydration and xylene clearing. Samples were embedded in paraffin and sectioned at 4 μm. Paraffin sections were deparaffinized with xylene and rehydrated through graded ethanol solutions before hematoxylin and eosin (H&E) staining. After staining, the sections were dehydrated through a graded ethanol series, cleared in xylene, mounted with neutral resin, and examined for histopathological evaluation under the microscope (Olympus, Tokyo, Japan).

### 2.6. Fecal Sampling, DNA Extraction, and 16S rRNA Library Preparation

At the experimental endpoint, fresh fecal pellets were collected from each mouse before anesthesia and euthanasia. Fecal samples were collected within the same time window to reduce potential effects from circadian rhythm and handling. Each mouse was temporarily transferred to an individual sterile 1000 mL plastic cup inside a biosafety cabinet. Freshly excreted fecal pellets were collected with sterilized forceps. A separate pair of sterile forceps was used for each mouse. The pellets were immediately transferred into sterile 1.5 mL cryotubes, frozen in liquid nitrogen, and kept at −80 °C before DNA extraction. Microbial genomic DNA was prepared with the FastPure Stool DNA Isolation Kit (MJYH, Shanghai, China). DNA quality was examined on a 1% agarose gel, and the concentration was measured using a NanoDrop 2000 spectrophotometer (Thermo Scientific, Waltham, MA, USA). The V3–V4 region of the bacterial 16S rRNA gene was amplified with barcoded 338F and 806R primers [12]. The PCR program included 27 cycles of denaturation at 95 °C for 30 s, annealing at 55 °C for 30 s, and extension at 72 °C for 45 s. PCR products were pooled, gel-purified, quantified, and libraries were prepared using the NEXTFLEX Rapid DNA-Seq Kit (Bioo Scientific, Austin, TX, USA). Paired-end sequencing was performed on an Illumina NextSeq 2000 PE300 platform (Illumina, San Diego, CA, USA).

### 2.7. 16S rRNA Sequencing

Raw reads were subjected to quality filtering using fastp (version 0.19.6) and merged using FLASH (version 1.2.11) [13,14]. Denoising into amplicon sequence variants (ASVs) was performed using DADA plugin in Qiime2 (version 2020.2), with chloroplast and mitochondrial sequences removed [15]. To eliminate the impact of sequencing depth differences, samples were flattened based on the minimum number of sequences. Taxonomic annotations of ASVs were based on the SILVA 16S rRNA database (version 138.2), and species annotations were performed using the Naïve Bayes classifier of QIIME2.

### 2.8. Bioinformatics Analysis

Alpha diversity indices were calculated at the ASV level after rarefaction. Beta diversity was evaluated using Bray–Curtis distances and visualized by principal coordinate analysis (PCoA) and non-metric multidimensional scaling (NMDS). Differences between groups were tested using permutational multivariate analysis of variance (PERMANOVA). Homogeneity of group dispersion was evaluated using PERMDISP based on the same Bray–Curtis distance matrix. Taxonomic composition at the phylum and genus levels was summarized using relative abundance profiles, and both shared and group-specific ASVs were assessed by Venn analysis. Differential taxa were identified using LEfSe (LDA > 2.0, *p* < 0.05, version 1.0.8).

### 2.9. Fecal Short-Chain Fatty Acid Analysis

Gas chromatography–mass spectrometry (GC–MS) was used to determine fecal short-chain fatty acid (SCFA) concentrations. Fecal samples were weighed and acidified with hydrochloric acid. The samples were then homogenized with distilled water and extracted with ethyl acetate. After extraction, the samples were concentrated under nitrogen. The residues were dissolved in mass spectrometry-grade anhydrous ethanol and centrifuged before GC–MS analysis. SCFA concentrations were calculated using external standard calibration curves and normalized to the initial fecal wet weight. Levels of individual SCFAs were compared between the GMF and HMF groups.

### 2.10. Statistical Analysis

Statistical analyses were performed using GraphPad Prism 8.0 and R 4.4. Unless otherwise specified, data are expressed as mean ± SD. Normally distributed data were analyzed using Student’s *t*-test, and non-normally distributed data were analyzed using Wilcoxon rank-sum tests. Tumor growth curves were analyzed using two-way repeated-measures ANOVA followed by Sidak’s multiple comparisons test. For comparisons of alpha-diversity indices and fecal SCFA concentrations between groups, as well as Spearman correlation analyses between microbial taxa and fecal SCFA concentrations, *p*-values were consistently adjusted using the Benjamini–Hochberg false discovery rate method. All tests were two-tailed, and a *p*-value < 0.05 was considered statistically significant, with significance levels expressed as * *p* < 0.05, ** *p* < 0.01, and *** *p* < 0.001, respectively.

## 3. Results

### 3.1. HMF Exposure Suppresses Subcutaneous LLC Tumor Growth

**Figure 1:**
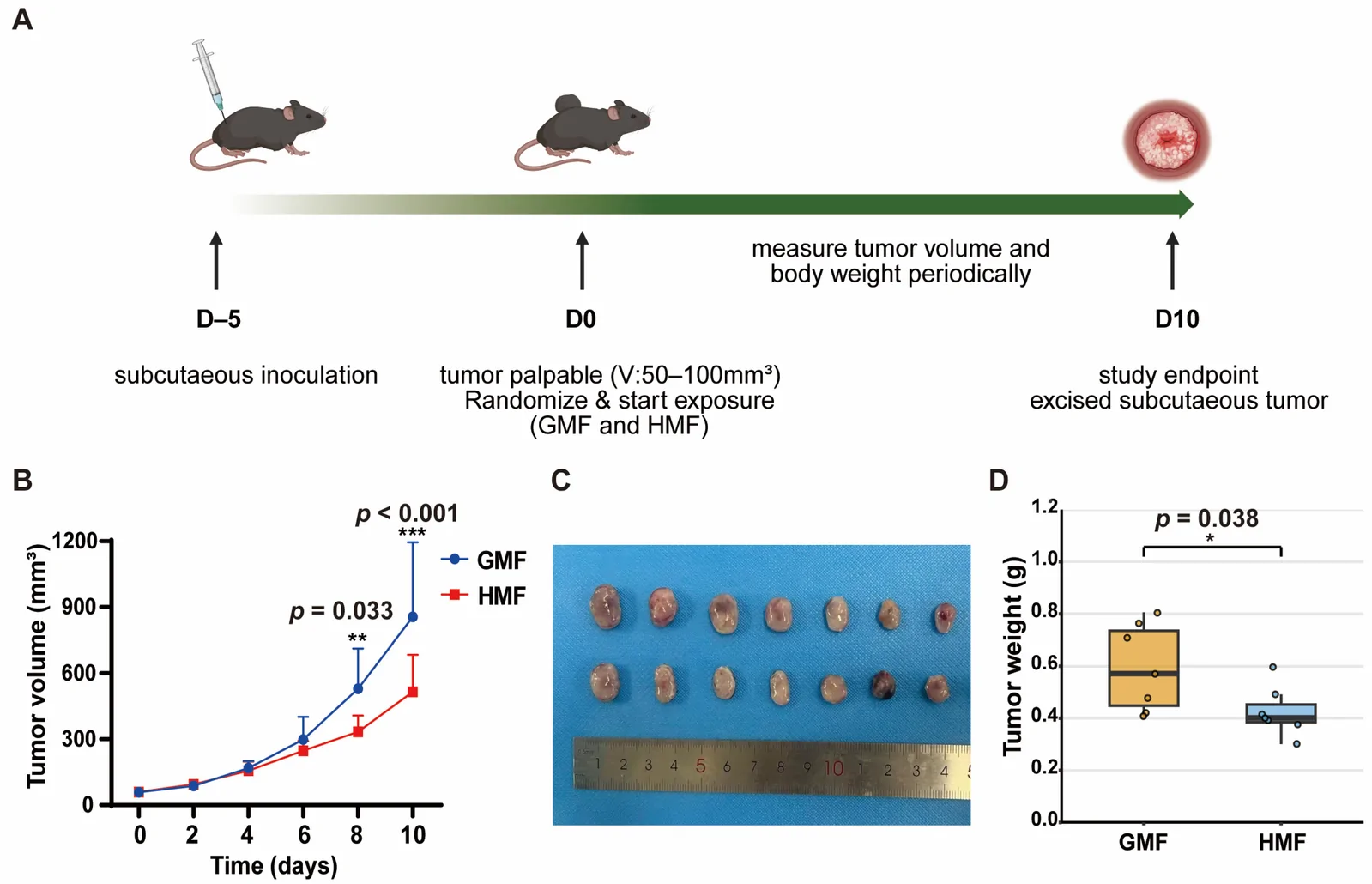
Hypomagnetic field exposure attenuates the growth of subcutaneous LLC tumors in C57BL/6 mice. (**A**) Experimental scheme and timeline. Created in BioRender. Mengmeng, L. (2026) https://BioRender.com/ox19bkg. (**B**) Tumor growth curves of the GMF and HMF groups over time. Tumor volumes were analyzed using two-way repeated-measures ANOVA (GMF versus HMF, F(1, 12) = 5.083, *p* = 0.044), followed by Sidak’s multiple comparisons test at each time point. HMF significantly reduced tumor volume on day 8 (*p* = 0.033) and day 10 (*p* < 0.001). (**C**) Excised subcutaneous tumor at endpoint. (**D**) Endpoint tumor weight comparison between groups (two-tailed unpaired *t*-test, *p*  = 0.038). Data are presented as mean  ±  SD (*n* = 7 mice per group). Statistical significance is indicated (* *p* < 0.05, ** *p* < 0.01, and *** *p* < 0.001).

Mice bearing LLC tumors were randomly assigned to either geomagnetic field (GMF) or hypomagnetic field (HMF) conditions once tumor volume reached approximately 50–100 mm^3^, and exposure was maintained until the experimental endpoint (Figure 1A). Throughout the exposure period, the growth trend of tumor volume in the HMF group was overall lower than that in the GMF group, suggesting that HMF exposure can attenuate the progression of tumors (Figure 1B). The tumor growth curves were analyzed using two-way repeated-measures ANOVA, with an effect size of partial eta-squared (partial η^2^ = 0.298). At the experimental endpoint, the mean tumor volume was lower in the HMF group than in the GMF group, with a mean difference of 340.1 mm^3^ between groups (95% CI: 154.4 to 525.7; Sidak-adjusted *p* < 0.001). Endpoint tumor size and weight were significantly lower in the HMF group than in the GMF group (Figure 1C,D), further confirming that HMF exposure inhibits LLC tumor growth.

### 3.2. Evaluation of Overt Systemic Toxicity After HMF Exposure in LLC Tumor-Bearing Mice

**Figure 2:**
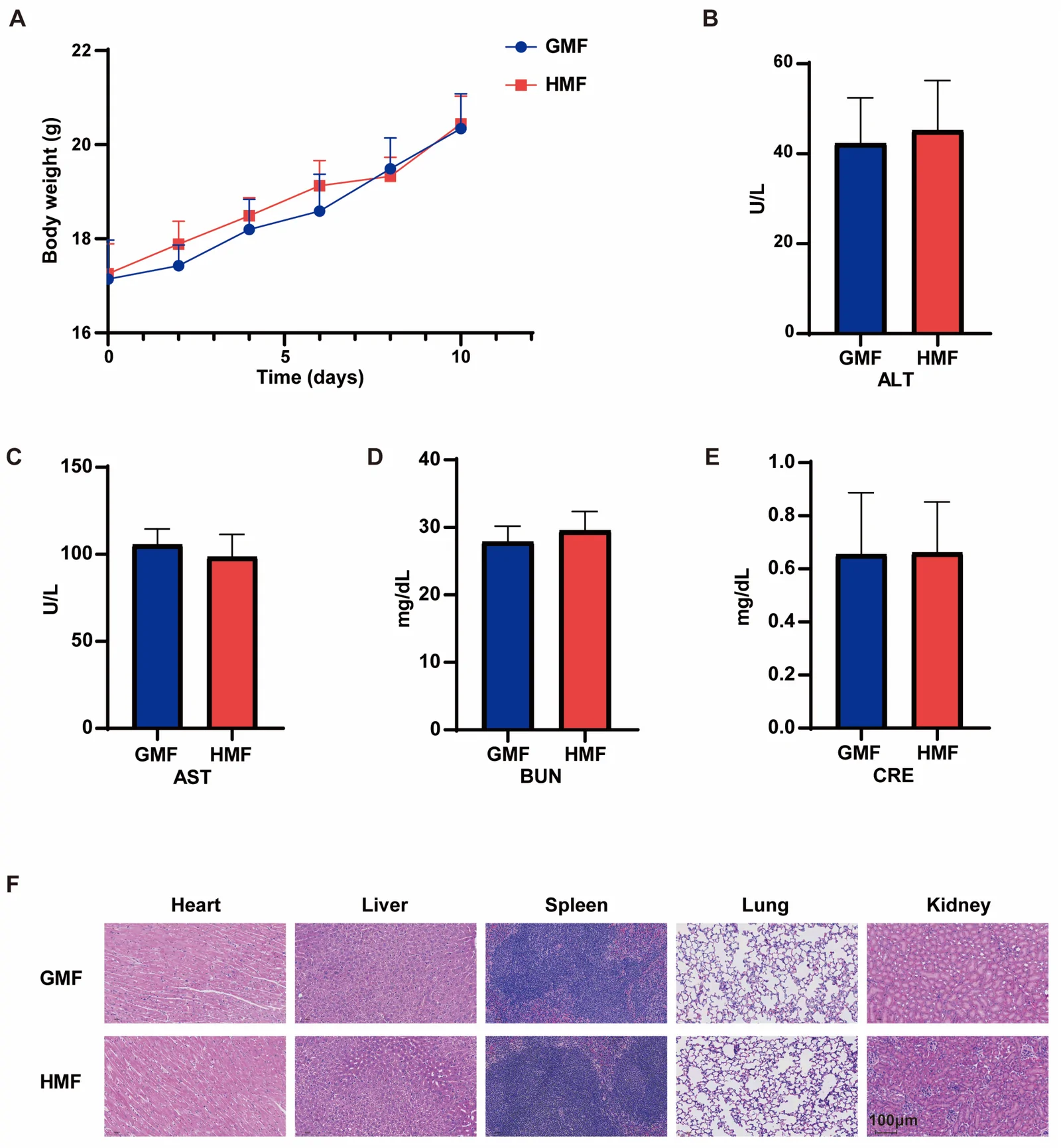
Assessment of systemic toxicity following HMF exposure. (**A**) Body weight during exposure (two-way repeated-measures ANOVA). (**B**) Serum ALT at endpoint (two-tailed unpaired *t* test). (**C**) Serum AST at endpoint (two-tailed unpaired *t* test). (**D**) Serum BUN at endpoint (two-tailed unpaired *t* test). (**E**) Serum creatinine at endpoint (two-tailed unpaired *t* test). (**F**) H&E staining of major organs at endpoint. Scale bars, 100 μm. Data are presented as mean ±  SD (*n* = 7 mice per group).

Body weight, selected serum biochemical markers, and histopathological changes in major organs were assessed to evaluate overt systemic toxicity in LLC tumor-bearing mice. During the experiment, both groups exhibited similar trends in body weight changes (Figure 2A). The levels of serum alanine aminotransferase (ALT) and aspartate aminotransferase (AST) were used as indicators of liver function, while the levels of blood urea nitrogen (BUN) and creatinine (CRE) were used as indicators of kidney function. No significant differences were observed in these parameters between the GMF and HMF groups (Figure 2B–E). Consistently, histopathological examination by H&E staining revealed no evident tissue damage in major organs, including the heart, liver, spleen, lung, and kidney (Figure 2F). These results indicate that, within the exposure intensity and time window of this study, HMF exposure did not cause any apparent systemic toxicity based on the measured parameters.

### 3.3. Assessment of Endpoint Gut Microbial Alpha Diversity in LLC Tumor-Bearing Mice Exposed to HMF

**Figure 3:**
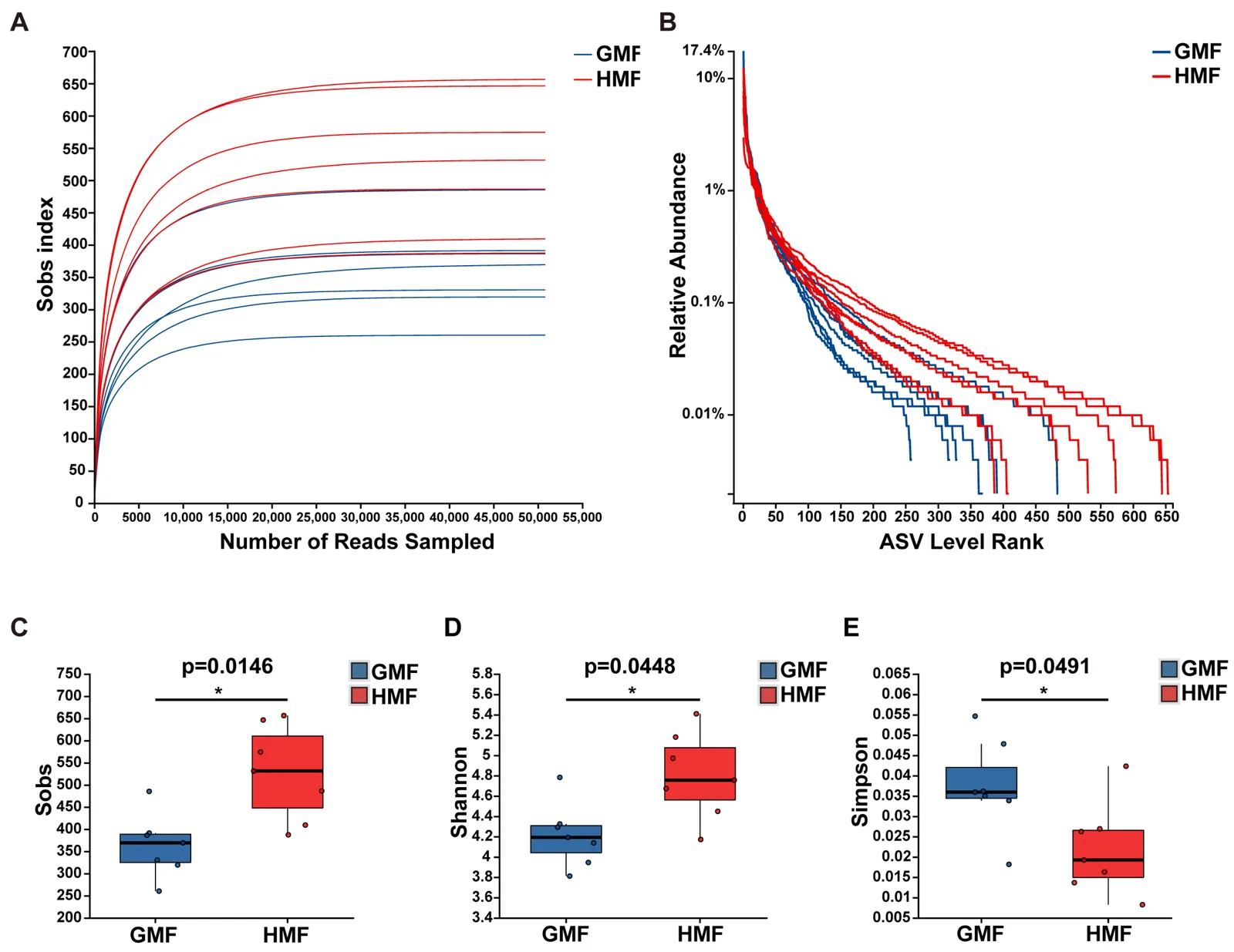
Sequencing depth assessment and alpha diversity of gut microbiota in the GMF and HMF groups. (**A**) Rarefaction curves. (**B**) Rank-abundance curves. (**C**–**E**) Alpha diversity (Sobs, Shannon, and Simpson, *n* = 7 per group). Statistical significance is indicated (* *p* < 0.05).

As shown in Figure 3A,B, the rarefaction curves for all samples reached a plateau, and the rank–abundance curves exhibited a monotonic decline in the relative abundance of ASVs. These results indicate that the sequencing depth was sufficient to capture the vast majority of microbial taxa, providing adequate coverage of the microbial community. Alpha diversity analysis based on ASV levels (Figure 3C) showed that the observed ASV richness (Sobs) in the HMF group was significantly higher than that in the GMF group (*p* = 0.0146). The Shannon index was significantly greater in the HMF group (*p* = 0.0448), while the Simpson index was significantly lower (*p* = 0.0491). These endpoint differences indicate that HMF exposure was associated with increased gut microbial richness and diversity compared with GMF controls.

### 3.4. Assessment of Endpoint Gut Microbial Beta Diversity in LLC Tumor-Bearing Mice Exposed to HMF

**Figure 4:**
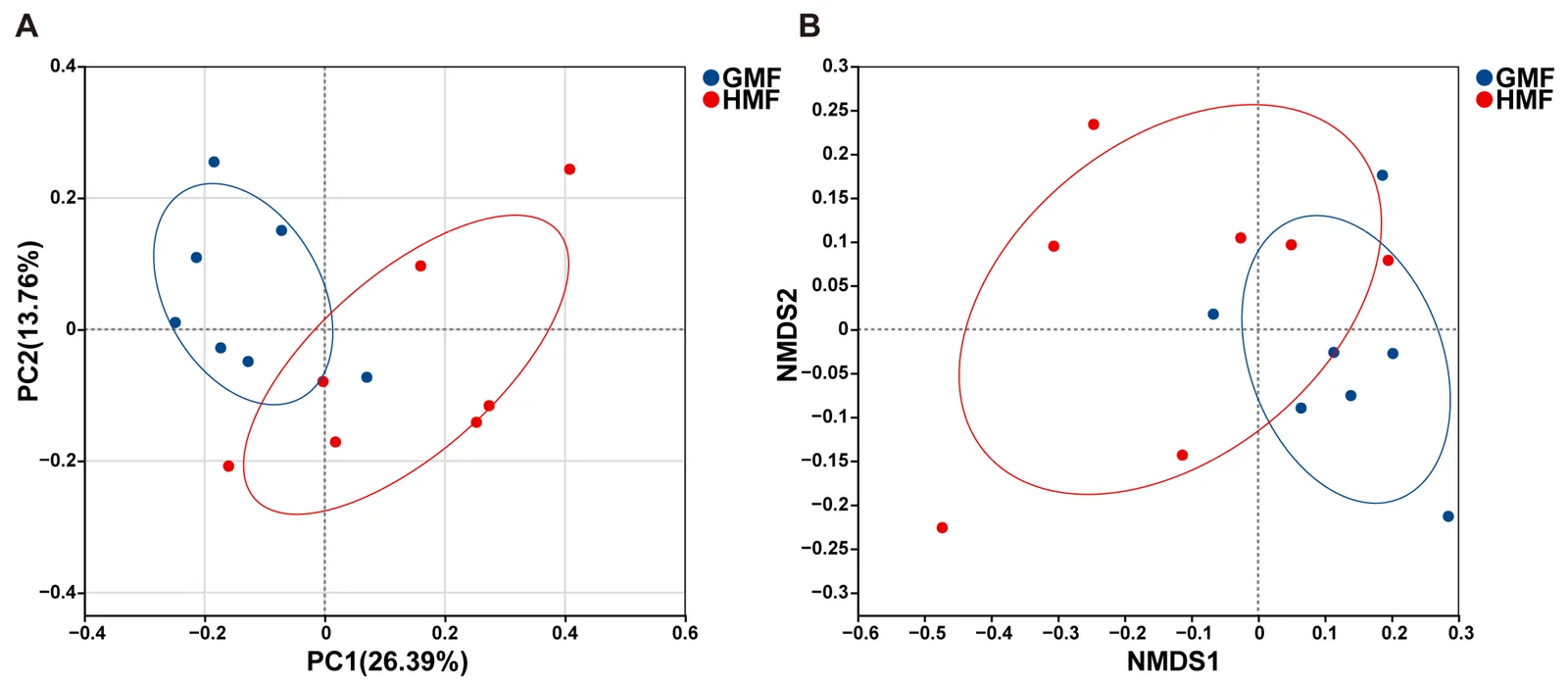
Beta diversity analysis at the ASV level. (**A**) PCoA analysis (*n* = 7 per group). (**B**) NMDS analysis (*n* = 7 per group).

To further evaluate overall variations in microbial community structure between the GMF and HMF groups, beta diversity analysis was performed at the ASV level using the Bray–Curtis distance metric. Principal Coordinate Analysis (PCoA) revealed that the first two principal coordinates (PC1 and PC2) explained 26.39% and 13.76% of the total variance, respectively (Figure 4A). Consistently, the NMDS ordination also suggested a separation trend between the GMF and HMF microbiota (Figure 4B). Furthermore, permutational multivariate analysis of variance (PERMANOVA) results confirmed that HMF exposure was associated with a significant difference in overall community composition compared with controls (R^2^ = 0.1799, *p* = 0.003; 999 permutations). PERMDISP analysis showed no statistically significant difference in dispersion between the two groups (F = 4.062, *p* > 0.05, 999 permutations).

### 3.5. Taxonomic Composition and Shared Features of the Gut Microbiota

**Figure 5:**
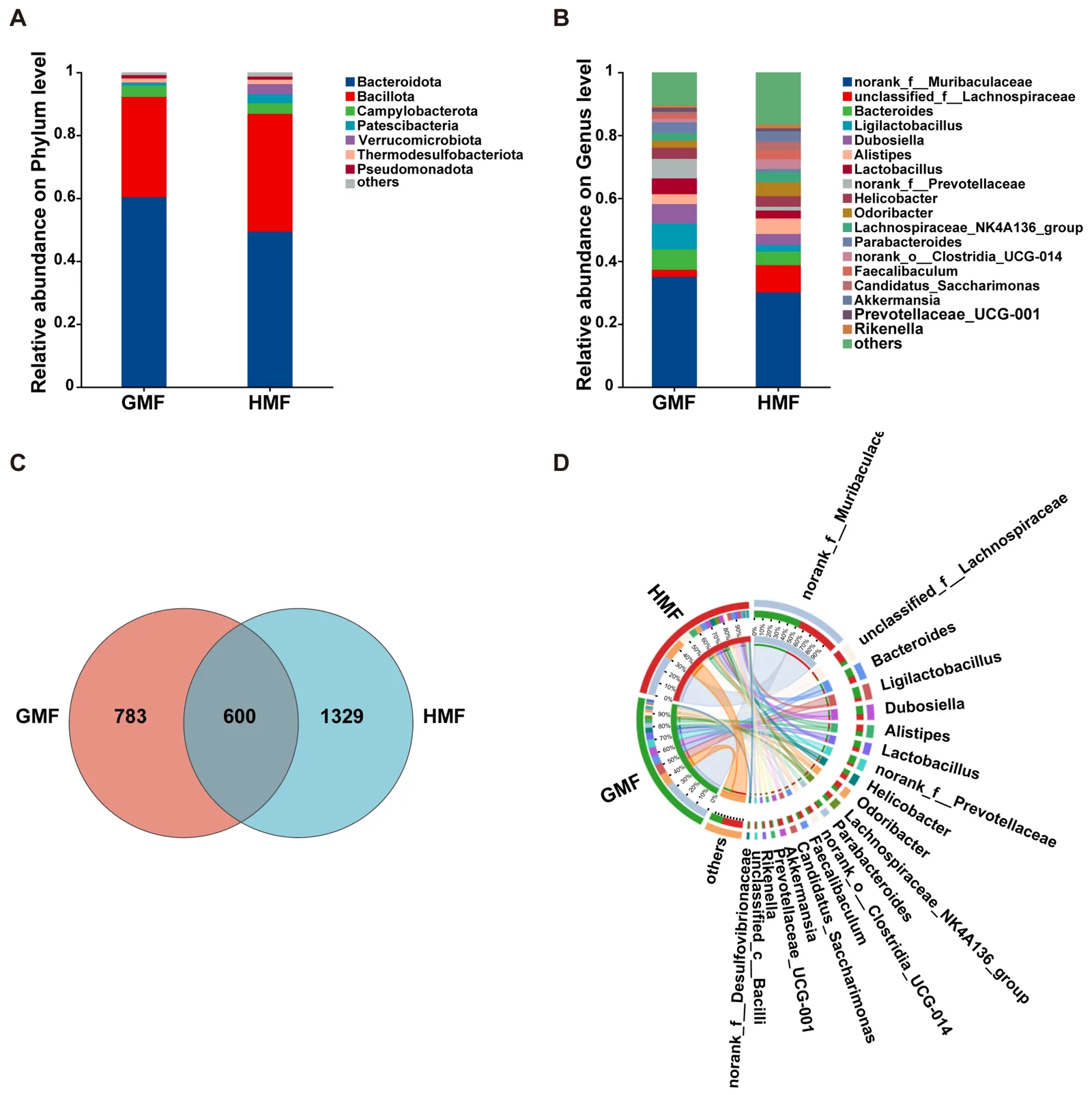
Taxonomic differences in the gut microbiota of LLC tumor-bearing mice. (**A**) Relative abundance at phylum level. (**B**) Relative abundance at genus level. (**C**) Venn diagram of shared and unique ASVs. (**D**) Circos plot of genus relationships.

We analyzed the phylum-level taxonomic composition in each group (Figure 5A). The communities in both groups were mainly composed of Bacteroidota and Bacillota, while Campylobacterota, Patescibacteria, Verrucomicrobiota, Thermodesulfobacteriota, and Pseudomonadota were present in lower proportions. The HMF group showed a modest decrease in Bacteroidota and a slight increase in Bacillota relative to the GMF group. At the genus level, both groups were mainly composed of norank_f_*_Muribaculaceae*, *Ligilactobacillus*, *Bacteroides*, *Dubosiella*, and *Lactobacillus*, together with several low-abundance genera (Figure 5B). Notably, the proportion of *unclassified_f__Lachnospiraceae* appeared higher in the HMF group, whereas *Bacteroides* and *Ligilactobacillus* were lower in HMF. To explore the differences in species overlap, we constructed a Venn diagram at the ASV level. Although the GMF group and the HMF group shared 600 ASVs, the HMF group had more unique ASVs detected (Figure 5C). In addition, genus-level differences were summarized using a circos plot (Figure 5D).

### 3.6. LEfSe-Based Identification of Taxa Differing Between Groups

**Figure 6:**
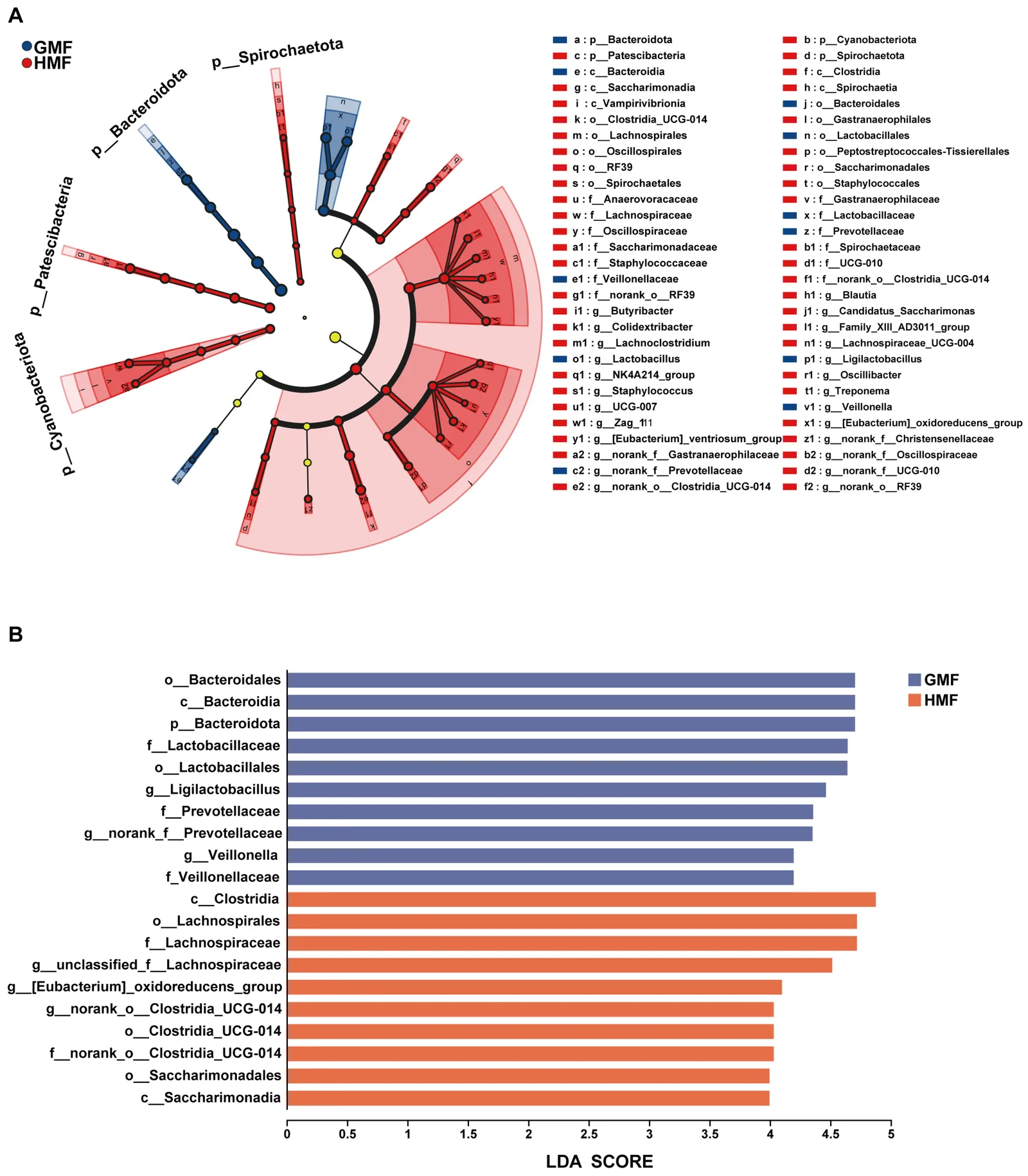
LEfSe-based identification of differential taxa between GMF and HMF groups. (**A**) LEfSe cladogram. (**B**) LDA score distribution.

LEfSe analysis was applied from the phylum to the genus level to identify bacterial taxa that contributed to the separation between the GMF and HMF groups, using an LDA score threshold of 2.0. The cladogram revealed several discriminative lineages between the two groups (Figure 6A). In the GMF group, enriched taxa were mainly assigned to the Bacteroidota lineage, including Bacteroidia and Bacteroidales, together with Lactobacillales/Lactobacillaceae members such as *Ligilactobacillus* and taxa within Prevotellaceae. In contrast, the HMF group was enriched in several Clostridia-related lineages, particularly Lachnospirales/Lachnospiraceae, including *unclassified_f__Lachnospiraceae* and *[Eubacterium]_oxidoreducens_group*, as well as *Clostridia_UCG-014*. Enrichment of Saccharimonadia/Saccharimonadales was also observed in the HMF group. The LDA score plot highlighted the taxa contributing most to group discrimination, with HMF exposure primarily associated with *Clostridia*, particularly the *Lachnospiraceae* family, whereas GMF controls were enriched in *Bacteroidota* and *Lactobacillales*-related taxa (Figure 6B). These LEfSe-identified genus-level differences were further evaluated using Wilcoxon rank-sum tests with Benjamini–Hochberg correction (Figure S2).

### 3.7. Fecal SCFA Profiles and Their Associations with Gut Microbiota

**Figure 7:**
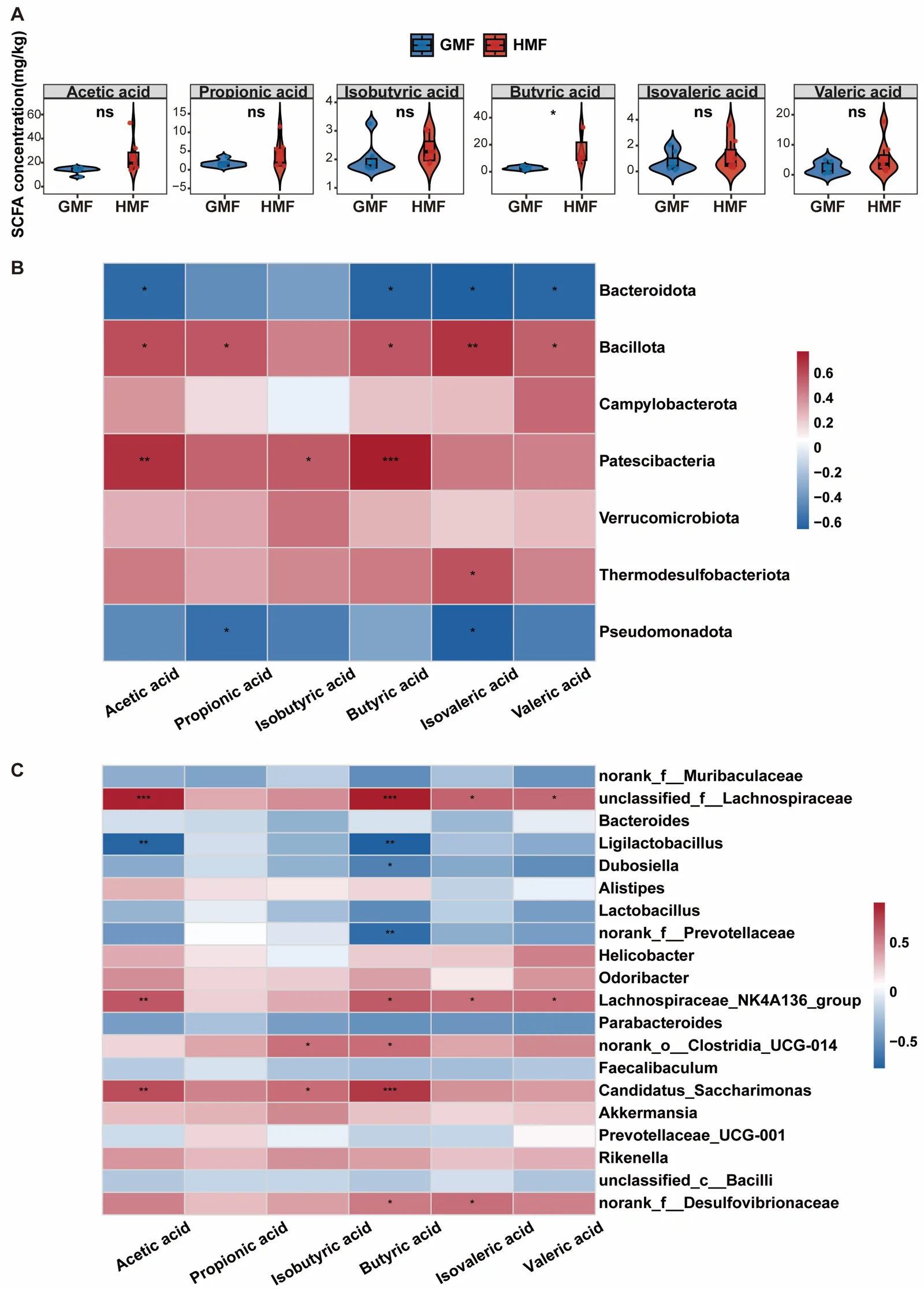
Alterations in fecal short-chain fatty acids and their microbial correlations. (**A**) SCFA concentration profiles (*n* = 7 per group). (**B**) Correlation heatmap of phylum-level taxa and SCFAs (*n* = 14). (**C**) Correlation heatmap of genus-level taxa and SCFAs (*n* = 14). Statistical significance is indicated (* *p* < 0.05, ** *p* < 0.01, and *** *p* < 0.001).

Fecal short-chain fatty acid (SCFA) analysis showed that HMF exposure altered the levels of several microbial metabolites. Among the six detected SCFAs, butyric acid was significantly elevated in the HMF group compared with the GMF group (GMF: 2.77 ± 1.23 mg/kg; HMF: 15.54 ± 12.03 mg/kg, *n* = 7 per group, *p* = 0.013). Acetic acid, propionic acid, isobutyric acid, isovaleric acid, and valeric acid showed no significant differences between groups (Figure 7A).

Associations between gut microbial taxa and fecal SCFA concentrations were further assessed using Spearman correlation analysis. At the phylum level, Bacillota showed positive associations with acetic, butyric, and valeric acids, whereas Bacteroidota was inversely associated with several SCFA parameters (Figure 7B). At the genus level, the analysis shown in Figure 7C was performed using the top 20 genera ranked by mean relative abundance across all samples. Lachnospiraceae-related taxa, including *unclassified_f__Lachnospiraceae* and *Lachnospiraceae_NK4A136_group*, were positively associated with several SCFAs, particularly acetic acid and butyric acid. *Clostridia_UCG-014*-related taxa also showed positive associations with SCFA levels. By contrast, *Ligilactobacillus*, *Lactobacillus*, *Bacteroides*, and *norank_f__Muribaculaceae* were negatively associated with multiple SCFAs. The results of the Spearman correlation analyses at the phylum and genus levels are provided in Supplementary Tables S1–S4. These results indicate that HMF-associated enrichment of Clostridia and Lachnospiraceae-related taxa was accompanied by altered fecal SCFA profiles, especially butyrate-related changes.

## 4. Discussion

In this study, we evaluated the effects of hypomagnetic field (HMF) exposure on subcutaneous Lewis lung carcinoma (LLC) progression in C57BL/6 mice and examined endpoint gut microbial and fecal SCFA profiles. HMF exposure suppressed subcutaneous LLC tumor growth without overt systemic toxicity based on the measured parameters. Endpoint 16S rRNA sequencing showed that HMF-exposed mice had higher gut microbial richness and diversity than GMF controls. Dissimilarity analysis further indicated differences in overall endpoint microbial community structure between the GMF and HMF groups. While broadly similar taxonomic profiles at the phylum and genus levels, the HMF group had a larger number of group-specific ASVs while maintaining the common core feature set. Fecal SCFA profiling showed group differences in microbial metabolite concentrations, among which butyric acid showed an evident increase in the HMF group. Correlation analyses showed associations between Bacillota-related taxa, including Lachnospiraceae-related taxa, and fecal SCFA levels, suggesting the potential relationship between endpoint microbial composition and fecal metabolite profiles. HMF exposure was associated with LLC tumor growth inhibition, endpoint differences in gut microbial composition, and altered fecal SCFA profiles.

In our model, exposure to an approximately 20 nT hypomagnetic field suppresses the progression of subcutaneous LLC tumors. The geomagnetic field represents a natural condition for living systems on Earth. The differences observed between GMF and HMF conditions may reflect biological responses to altered magnetic environments rather than a simple inhibitory effect caused by HMF alone. Similar inhibitory effects on tumor cell proliferation and tumor growth have been reported under different magnetic exposure conditions. However, biological responses to magnetic field exposure are highly dependent on exposure parameters and biological contexts. Variations in field intensity, frequency, waveform, exposure duration, and tumor model have been associated with divergent outcomes, with both tumor-inhibitory and tumor-promoting effects reported in previous studies [16,17]. For instance, low-frequency magnetic fields (LF-MFs) have been shown to inhibit tumor growth in LLC-bearing mice, potentially through mechanisms involving suppression of cellular iron metabolism, stabilization of the p53 protein, and activation of the p53-miR-34a-E2F1/E2F3 signaling axis [18]. In addition, hypomagnetic conditions (~0.4–1 μT) were reported to inhibit the growth of transplanted fibrosarcoma and pancreatic tumors. Nevertheless, given the limited sample size in that pilot study, the inhibitory effect did not reach statistical significance in the fibrosarcoma model [19]. Conversely, tumor-promoting responses have also been reported under certain exposure windows. In HT-1080 human fibrosarcoma cells, weak static fields with superimposed extremely low-frequency magnetic fields produced frequency-dependent bidirectional effects, where narrow frequency shifts were sufficient to switch the response from inhibition to significant growth acceleration [20]. Our study demonstrates that tumor growth is modulated under a near-zero magnetic environment (~20 nT), extending previous studies of hypomagnetic effects to lower magnetic intensities.

Several mechanisms may contribute to the reduced tumor growth observed under HMF exposure. Previous studies have suggested that altered magnetic environments may influence cellular processes involved in stress adaptation and metabolic regulation. HMF exposure has been reported to affect intracellular ROS levels and mitochondrial oxidative stress, both of which are closely related to tumor cell proliferation [21]. In addition, HMF exposure has been shown to perturb circadian rhythms and associated signaling pathways, particularly those involving cryptochromes [22]. Since these clock proteins are important regulators of cell proliferation and metabolism, their dysregulation may alter tumor cell behavior. Non-ionizing magnetic fields have also been shown to activate cellular stress responses such as autophagy and related signaling cascades, thereby altering the state of the cells [23].

The mechanisms underlying microbiota changes under near-zero magnetic conditions remain unclear. HMF exposure may affect the gut microbiota indirectly by altering host conditions that influence the intestinal environment. These host responses may contribute to the differences in gut microbial composition observed in HMF-exposed tumor-bearing mice. Direct effects of magnetic field attenuation on microbial growth or metabolism are also possible. Some microorganisms contain redox-active enzymes and iron-associated proteins that could be sensitive to changes in magnetic conditions [24,25].

The lack of obvious systemic toxicity after HMF exposure provides important context for the microbiome findings. Severe physiological stress, cachexia, or organ injury can independently reshape the gut microbiota [26,27]. In this study, body weight, serum biochemical indices, and histological examination did not indicate overt systemic injury, suggesting that the microbiota differences in the HMF group were unlikely to be solely explained by general morbidity or organ damage. These changes may instead reflect the responses of the host–microbiota system to the altered magnetic environment. In this context, host-mediated factors such as oxidative status, circadian regulation, immune signaling, intestinal barrier function, and nutrient availability may shape the intestinal microenvironment and thereby influence microbial community structure [28,29].

Gut microbial diversity, particularly alpha diversity, is commonly regarded as a key indicator of intestinal ecosystem status. Lower alpha diversity has been reported in gut dysbiosis and in several chronic disease conditions [30]. In the present study, HMF exposure altered alpha diversity in LLC-bearing mice, with differences in microbial richness and evenness compared with GMF controls. This finding contrasts with previous observations in healthy mice, in which long-term (8–12 weeks) HMF exposure did not produce significant changes in alpha diversity [10]. The discrepancy may be related to host condition. Tumor-bearing mice have altered systemic and intestinal states, which may make the gut microbiota more responsive to external physical factors. It is also possible that HMF exposure influenced tumor-associated microbial patterns, although this interpretation requires further functional evidence. The increased richness observed under approximately 20 nT HMF is also similar to findings from mice exposed to an ultra-strong magnetic field (16 T), in which Shannon and Chao indices were increased [31]. These observations suggest that the effect of magnetic-field exposure on gut microbial diversity may depend on both field intensity and host status. Further studies are needed to determine whether the increase in alpha diversity contributes to changes in microbial metabolism or tumor progression.

Differences in overall microbial composition were also observed between the two groups. PCoA and NMDS plots showed partial separation between groups, and PERMANOVA confirmed a significant difference in community composition. HMF exposure appears to have shifted the gut microbial community to some extent, without producing a major restructuring of the whole community. A similar pattern has been reported in long-term HMF exposure studies, where significant alterations in beta diversity were observed after 8–12 weeks. Notably, these microbial changes were found to be reversible following a 4-week restoration of the geomagnetic field (RGMF) [10]. This reversibility indicates that HMF-induced microbial shifts may reflect adaptive ecological adjustments rather than persistent dysbiosis.

Gut microbial alterations under HMF exposure were associated with changes in microbial metabolite profiles. LEfSe analysis identified several enriched taxa in the HMF group, including members with potential metabolic relevance. Among these, members of the family Lachnospiraceae were significantly enriched. Lachnospiraceae are widely recognized as one of the major butyrate-producing bacterial groups in the gut [32]. Consistent with this taxonomic shift, fecal SCFA profiling showed that butyric acid exhibited a significant increase in the HMF group. In addition, genus-level correlation analysis showed positive associations between Lachnospiraceae-related taxa and several SCFAs, particularly acetic acid and butyric acid. These findings suggest that HMF-associated enrichment of Lachnospiraceae-related taxa may be accompanied by changes in SCFA metabolism. Butyrate is a key microbial metabolite involved in intestinal homeostasis and immune regulation. Previous studies have shown that butyrate can participate in tumor regulation through multiple mechanisms. It acts as a histone deacetylase (HDAC) inhibitor and can induce apoptosis in cancer cells [33]. It also strengthens intestinal epithelial barrier function, thereby reducing the translocation of proinflammatory endotoxins [34]. In addition, butyrate modulates immune cell differentiation and influences regulatory T cells (Tregs) and CD8^+^ T cell-mediated immune surveillance [35,36]. Compared with healthy individuals, patients with non-small cell lung cancer (NSCLC) have been reported to show reduced abundance of certain beneficial butyrate-producing bacteria, including members related to Lachnospiraceae. The enrichment of Clostridia_UCG-014 is also worth noting. Members of the Clostridia class are known for their ability to produce SCFA and to support Treg induction and immune modulation in the gut [37]. The enrichment of Lachnospiraceae-related taxa and Clostridia_UCG-014-related lineages, together with increased fecal butyrate levels, suggests a potential association between HMF-related microbial shifts and SCFA profiles.

In contrast, several bacterial taxa showed decreased abundance under HMF exposure. Bacteroides species are common gut commensals involved in polysaccharide metabolism, but their roles may differ among species and depend on host status and tumor-associated intestinal conditions. Some Bacteroides members have been linked to proinflammatory signaling and tumor-promoting contexts under specific conditions [38,39]. Lactobacillus abundance was also lower in the HMF group. Although Lactobacillus species are often considered beneficial, their effects may vary with microbial composition and host conditions. The reduction in Lactobacillus abundance has been reported in mice exposed to an ultra-strong static magnetic field (16 T), suggesting that magnetic field exposure may influence selected lactic acid bacterial populations [31]. These findings indicate that HMF exposure was related to changes in selected gut bacterial taxa, while the functional relevance of these changes requires interpretation in the context of host status and the broader microbial community.

However, this study also has several limitations. First, the sample size was relatively limited, particularly for 16S rRNA sequencing and fecal SCFA analysis, and the observed differences should be confirmed in larger cohorts. Second, a sham-shielded chamber or equivalent enclosure control was not included. Although temperature, humidity, light/dark schedule, ventilation, cage handling, and sampling procedures were standardized between groups, chamber-related environmental factors cannot be completely excluded as potential confounders. Future studies using a sham chamber or magnetic field restoration controls will be needed to further separate the effects of magnetic field attenuation from chamber-related effects. Third, the findings establish associations rather than a definitive causal link among HMF exposure, endpoint microbiota differences, SCFA alterations, and tumor growth inhibition. Because fecal microbiota was assessed only at the experimental endpoint, the observed microbiota differences may reflect a combination of HMF exposure, tumor-related changes, and host physiological responses. Future studies incorporating baseline fecal sampling, mice exposed to HMF without tumor, fecal microbiota transplantation, antibiotic depletion, and SCFA supplementation will be needed to determine whether HMF-associated microbiota or SCFA changes directly contribute to tumor growth inhibition. Fourth, although fecal SCFAs were measured, serum SCFA levels, intestinal barrier markers, tumor-infiltrating lymphocytes, and tumor immune microenvironmental changes were not assessed. These factors should be evaluated in future studies to clarify the host-mediated mechanisms potentially involved in the response to HMF exposure. Finally, this study utilized a single HMF intensity and a fixed exposure duration. Exploring dose–response relationships across magnetic parameters will be important for understanding the biological effects of HMF exposure. Future studies incorporating additional magnetic field intensities, longer observation periods, and complementary mechanistic analyses may help clarify how altered magnetic field conditions influence tumor biology and host-associated microbial features.

## 5. Conclusions

Overall, HMF exposure suppressed subcutaneous LLC tumor growth without evident systemic toxicity. This tumor phenotype was accompanied by increased gut microbial richness, a statistically significant but limited shift in community structure, and changes in fecal SCFA profiles. HMF exposure did not cause broad disruption of the dominant microbial community, but was associated with increased group-specific ASVs. Among the measured SCFAs, butyric acid showed significant increase in the HMF group. These findings indicate that near-zero magnetic field exposure is associated with changes in tumor growth, gut microbial composition, and microbial metabolites. However, the present data do not establish that microbiota alterations or butyrate mediated the tumor-suppressive effect of HMF exposure. Further studies require direct intervention of the microbiota or SCFAs, together with immune and metabolic analyses to determine whether the observed microbiota and SCFA changes contribute directly to the tumor phenotype under HMF exposure.

## Data Availability

The 16S rRNA sequencing data have been deposited in the NCBI Sequence Read Archive (SRA) under BioProject accession number PRJNA1431342. The data will be publicly available upon publication. Reviewer access is available upon request. Other data that support the findings of this study are available from the corresponding author upon request.

## Supplementary Materials

The following supporting information can be downloaded at: https://www.mdpi.com/article/10.3390/biology15161349/s1. Figure S1. Magnetic shielding device and field distribution in the working region. (A) Internal environment of the HMF chamber. (B) Magnetic field intensity heatmaps of the working region under light-off conditions (upper panel) and light-on conditions (lower panel). Figure S2. Differential bacterial genera between GMF and HMF groups identified by Wilcoxon rank-sum test. Statistical significance is indicated (* *p*  < 0.05). Table S1. Spearman rho values for correlations between phylum-level taxa and SCFAs. Table S2. Benjamini–Hochberg-adjusted *p* values for correlations between phylum-level taxa and SCFAs. Table S3. Spearman rho values for correlations between genus-level taxa and SCFAs. Table S4. Benjamini–Hochberg-adjusted *p* values for correlations between genus-level taxa and SCFAs.

## Abbreviations

The following abbreviations are used in this manuscript:

HMF	Hypomagnetic field
GMF	Geomagnetic field
LLC	Lewis lung carcinoma
ALT	Alanine aminotransferase
AST	Aspartate aminotransferase
BUN	Blood urea nitrogen
CRE	Creatinine
ASV	Amplicon sequence variants
PCoA	Principal coordinate analysis
NMDS	Non-metric multidimensional scaling
PERMANOVA	Permutational multivariate analysis of variance
SCFA	Short-chain fatty acid
GC–MS	Gas chromatography–mass spectrometry
HDAC	Histone deacetylase
Tregs	Regulatory T cells
NSCLC	Non-small cell lung cancer
FMT	Fecal microbiota transplantation
TILs	Tumor-infiltrating lymphocytes
