# Supplementary material for: Effects of Hypomagnetic Field Environment on Tumor Progression and Gut Microbiota in LLC Tumor-Bearing Mice
Source: Biology (Basel). 2026 Aug 10;15(16):1349. doi: 10.3390/biology15161349 (PMC13509595; doi:10.3390/biology15161349)
Supplement: Supplementary file 1 [file biology-15-01349-s001.zip › biology-4422959-supplementary.pdf]

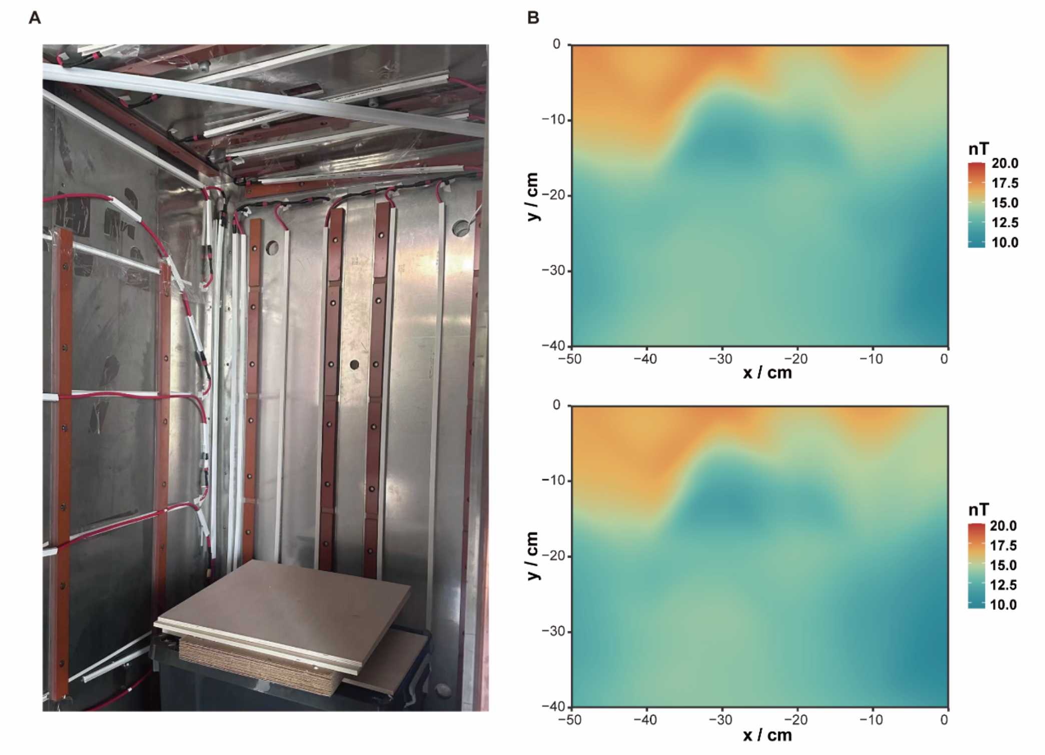

Figure S1. Magnetic shielding device and field distribution in the working region. (A) Internal environment of the HMF chamber. (B) Magnetic field intensity heatmaps of the working region under light-off conditions (upper panel) and light-on conditions (lower panel).

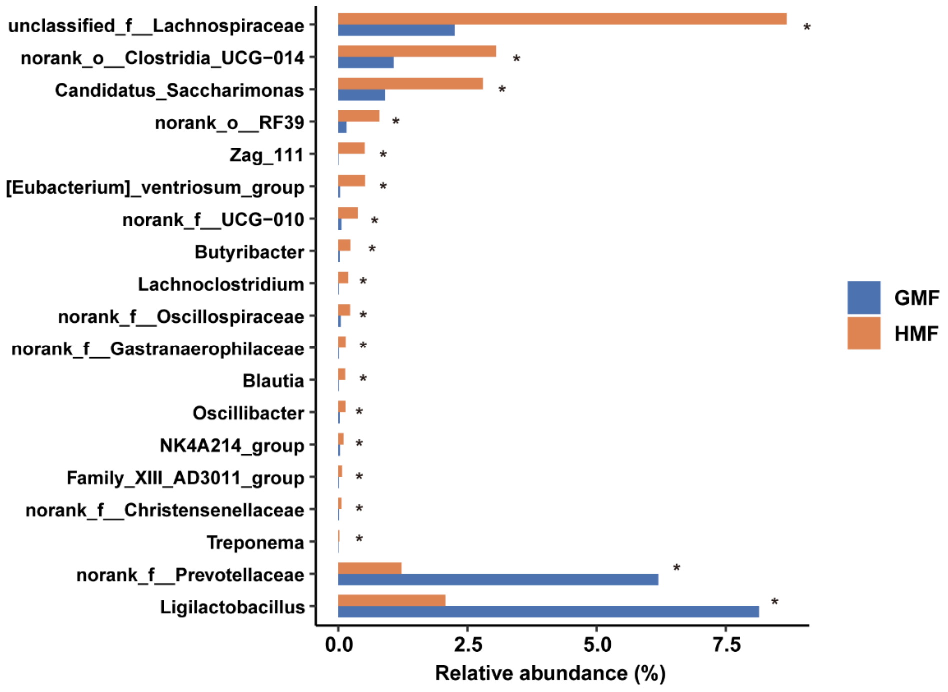

Figure S2. Differential bacterial genera between GMF and HMF groups identified by Wilcoxon rank-sum test. Statistical significance is indicated (*$p < 0.05$).

Table S1. Spearman rho values for correlations between phylum-level taxa and SCFAs

Phylum	Acetic acid	Propionic acid	Isobutyric acid	Butyric acid	Isovaleric acid	Valeric acid
Bacteroidota	-0.604	-0.429	-0.349	-0.626	-0.648	-0.613
Bacillota	0.609	0.578	0.446	0.578	0.692	0.543
Campylobacterota	0.367	0.169	0.002	0.240	0.257	0.521
Patescibacteria	0.697	0.530	0.565	0.780	0.455	0.437
Verrucomicrobiota	0.304	0.323	0.484	0.286	0.216	0.264
Thermodesulfobacteriota	0.464	0.327	0.415	0.464	0.591	0.433
Pseudomonadota	-0.446	-0.569	-0.499	-0.332	-0.648	-0.495

Table S2. Benjamini–Hochberg-adjusted *p* values for correlations between phylum-level taxa and SCFAs

Phylum	Acetic acid	Propionic acid	Isobutyric acid	Butyric acid	Isovaleric acid	Valeric acid
Bacteroidota	0.022	0.126	0.221	0.017	0.012	0.020
Bacillota	0.021	0.030	0.110	0.030	0.006	0.045
Campylobacterota	0.197	0.563	0.994	0.409	0.375	0.056
Patescibacteria	0.006	0.051	0.035	<0.001	0.102	0.118
Verrucomicrobiota	0.291	0.259	0.079	0.322	0.459	0.362
Thermodesulfobacteriota	0.095	0.253	0.140	0.095	0.026	0.122
Pseudomonadota	0.110	0.034	0.069	0.246	0.012	0.072

Table S3. Spearman rho values for correlations between genus-level taxa and SCFAs

**Genus**	**Acetic acid**	**Propionic acid**	**Isobutyric acid**	**Butyric acid**	**Isovaleric acid**	**Valeric acid**
norank_f__Muribaculaceae	-0.341	-0.385	-0.174	-0.503	-0.240	-0.468
unclassified_f__ Lachnospiraceae	0.864	0.367	0.459	0.908	0.622	0.604
Bacteroides	-0.081	-0.121	-0.327	-0.068	-0.284	-0.024
Ligilactobacillus	-0.749	-0.081	-0.323	-0.767	-0.240	-0.363
Dubosiella	-0.363	-0.103	-0.327	-0.574	-0.376	-0.503
Alistipes	0.327	0.174	0.143	0.218	-0.152	-0.007
Lactobacillus	-0.314	-0.029	-0.257	-0.530	-0.174	-0.420
norank_f__Prevotellaceae	-0.455	0.055	-0.055	-0.692	-0.332	-0.420
Helicobacter	0.367	0.169	0.002	0.240	0.257	0.521
Odoribacter	0.468	0.213	0.248	0.389	0.143	0.424
Lachnospiraceae_NK4-A136_group	0.670	0.235	0.363	0.648	0.560	0.560
Parabacteroides	-0.425	-0.238	-0.427	-0.497	-0.480	-0.495
norank_o__Clostridia_ UCG-014	0.209	0.376	0.569	0.587	0.376	0.473
Faecalibaculum	-0.191	-0.064	-0.222	-0.262	-0.253	-0.209
Candidatus_Saccharimonas	0.705	0.490	0.582	0.785	0.442	0.420
Akkermansia	0.304	0.323	0.484	0.286	0.216	0.264
Prevotellaceae_UCG-001	-0.112	0.204	-0.002	-0.156	-0.147	0.095
Rikenella	0.437	0.314	0.455	0.389	0.284	0.354
unclassified_c__Bacilli	-0.200	-0.147	-0.143	-0.209	-0.090	-0.218
norank_f__ Desulfovibrionaceae	0.516	0.301	0.389	0.538	0.582	0.508

Table S4. Benjamini–Hochberg-adjusted p values for correlations between genus-level taxa and SCFAs

**Genus**	**Acetic acid**	**Propionic acid**	**Isobutyric acid**	**Butyric acid**	**Isovaleric acid**	**Valeric acid**
norank_f__Muribaculaceae	0.233	0.175	0.553	0.067	0.409	0.091
unclassified_f__ Lachnospiraceae	<0.001	0.197	0.098	<0.001	0.018	0.022
Bacteroides	0.782	0.681	0.253	0.817	0.326	0.935
Ligilactobacillus	0.002	0.782	0.260	0.001	0.409	0.203
Dubosiella	0.203	0.725	0.253	0.032	0.185	0.067
Alistipes	0.253	0.553	0.626	0.455	0.605	0.982
Lactobacillus	0.274	0.923	0.375	0.051	0.553	0.135
norank_f__Prevotellaceae	0.102	0.852	0.852	0.006	0.246	0.135
Helicobacter	0.197	0.563	0.994	0.409	0.375	0.056
Odoribacter	0.091	0.464	0.392	0.169	0.626	0.131
Lachnospiraceae_NK4-A136_group	0.009	0.418	0.203	0.012	0.037	0.037
Parabacteroides	0.130	0.413	0.128	0.070	0.083	0.072
norank_o__Clostridia_ UCG-014	0.474	0.185	0.034	0.027	0.185	0.088
Faecalibaculum	0.513	0.829	0.446	0.366	0.383	0.474
Candidatus_Saccharimonas	0.005	0.075	0.029	<0.001	0.114	0.135
Akkermansia	0.291	0.259	0.079	0.322	0.459	0.362
Prevotellaceae_UCG-001	0.703	0.483	0.994	0.594	0.615	0.748
Rikenella	0.118	0.274	0.102	0.169	0.326	0.215
unclassified_c__Bacilli	0.493	0.615	0.626	0.474	0.759	0.455
norank_f__ Desulfovibrionaceae	0.059	0.296	0.169	0.047	0.029	0.064
